# Lactic Acidosis in the Presence of Glucose Diminishes Warburg Effect in Lung Adenocarcinoma Cells

**DOI:** 10.3389/fonc.2020.00807

**Published:** 2020-06-12

**Authors:** Heriberto Prado-Garcia, Andrea Campa-Higareda, Susana Romero-Garcia

**Affiliations:** Department of Chronic-Degenerative Diseases, National Institute of Respiratory Diseases “Ismael Cosío Villegas”, Mexico City, Mexico

**Keywords:** tumor metabolic shift, tumor metabolic symbiosis, mitochondrial function, aerobic glycolysis, oxidative metabolism

## Abstract

Lactic acidosis (3 to 40 mM, pH < 6.9) is a condition found in solid tumors because tumor cells have a high rate of glucose consumption and lactate production even in the presence of oxygen; nevertheless, the microenvironment might still provide a sufficient glucose supply. Lactic acidosis has been proposed to shift metabolism from aerobic glycolysis toward oxidative phosphorylation (OXPHOS). We tested if lung tumor cells cultured under lactic acidosis shift their metabolism from glycolysis to OXPHOS by consuming extracellular lactate, increasing growth rate. We analyzed lung adenocarcinoma (A-549, A-427) cell lines and non-transformed fibroblast cells (MRC-5), which were cultured using RPMI-1640 medium initially containing lactate (2 mM) and glucose (10 mM), at pH 7.2 or 6.2 and oxygen tension 21% O_2_ (normoxia) or 2% O_2_ (hypoxia). We obtained growth curves, as well as glucose consumption and lactate production rates (measured during exponential growth) for each cell line. HIF-1α (Hypoxia-inducible factor 1 α), CS (citrate synthase) and AMPK (AMP-activated protein kinase) transcript levels were analyzed using RT-qPCR. By flow cytometry, we determined: (a) expression of glucose transporters (GLUT)1 and 4; (b) lactate transporters (MCT)1 and 4; (c) cell cycle profile, and (d) protein levels of HIF-1α, total and phosphorylated AMPK (pAMPK). Mitochondrial functionality was evaluated by measuring O_2_ consumption in tumor cells using polarography and a Clark-type electrode. Tumor and non-transformed cells used both aerobic glycolysis and OXPHOS for obtaining energy. As of 48 h of culture, lactate levels ranged from (4.5–14 mM), thus forming a lactic environment. Lactic acidosis diminished GLUT1/GLUT4 expression and glucose consumption in A-549, but not in A-427 cells, and induced differential expression of HIF-1α, AMPK, and CS transcripts. A-427 cells increased pAMPK and HIF-1α levels and shifted their metabolism increasing OXPHOS; thus supporting cell growth. Conversely, A-549 cells increased HIF-1α protein levels, but did not activate AMPK and diminished OXPHOS. A-549 cells survived by arresting cells in G1-phase. Our findings show that lactic acidosis diminishes Warburg effect in tumor cells, but this change does not necessarily promote a shift to OXPHOS. Hence, lung adenocarcinomas show a differential metabolic response even when they are under the same microenvironmental conditions.

## Introduction

In solid tumors, lactate concentrations range from 3 to 40 mM and, intratumoral pH can be as low as 6.0 (1). Also, intratumoral glucose levels are estimated in a proportion of 1:40 with respect to plasma levels; thus, lactic acidosis along with glucose availability is a condition that can be found in the tumor microenvironment (2). This condition is the result of metabolic reprogramming, where tumor cells increase glycolysis and produce lactate from pyruvate even under oxygen availability (Warburg effect). Lactate can be also produced from the metabolism of glutamine through glutaminolysis (3). Lactate can be exported out of the cell using the monocarboxylate-4 transporter (MCT4), while MCT1 can facilitate both its import and export depending on the pH gradient (4, 5).

It has been reported that lactic acidosis has a key role in malignant development through different mechanisms (6–8). One of such mechanisms is through supporting the metabolic shift; for instance, breast tumor cells (4T1 cells) under lactic acidosis diminish aerobic glycolysis and show a non-glycolytic phenotype, characterized by a high oxygen consumption rate. In contrast, in the absence of lactic acidosis, 4T1 tumor cells exhibit a high glycolytic rate (Warburg effect) (6). Another report indicates that different tumor cell lines are able to revert from Warburg effect into OXPHOS when they are exposed to lactic acidosis (20 mM and pH 6.7) (7). In this report, the metabolic shift was proved by determining the quantity of ATP produced by tumor cells using both pathways: glycolysis and OXPHOS. While under lactic acidosis, glycolysis and OXPHOS provide 13.4–5.7% and 86.6–94.3 of total ATP, respectively; without lactic acidosis, glycolysis and OXPHOS generate 52.2–23.7% and 47.8–76.3%, respectively (7). But some questions remain such as which is the carbon source that tumor cells use under lactic acidosis in the presence of glucose? And how does this shift affect tumor growth?

Interestingly, this metabolic shift from aerobic glycolysis to OXPHOS in the presence of lactic acidosis should be supporting tumor metabolic symbiosis. In this phenomenon, tumor cells localized in the vicinity of blood vessels shift their metabolism toward a more oxidative metabolism and use lactate for feeding Krebs cycle in spite of the presence of glucose. This behavior allows tumor cells localized away from blood vessels to have access to glucose and produce lactate (9). When tumor metabolic symbiosis was described in breast cancer, it was shown that normoxic cells expressed MCT1 to import lactate and perform OXPHOS, and the amount of glucose uptake was lower in MCT1-positive cells than in hypoxic MCT4-positive cells that consumed more glucose and produced lactate (10). In this context, the expression of glucose and lactate transporters in tumor cells under lactic acidosis becomes crucial in the tumor metabolic symbiosis. It has been reported that glucose transporter (GLUT1) expression is upregulated in tumor cells under hypoxia but lactic acidosis suppresses the hypoxic induction of the transporter (11). GLUT4 can be expressed in insulin-sensitive tissues, fat and muscle, GLUT4 can also be found in 6.6% of lung carcinomas, whereas GLUT1 is expressed in 74% of lung-carcinomas samples (12). Nonetheless, there are no reports that show how expression levels of GLUT4 are regulated in tumor cells cultured under lactic acidosis. Regarding lactate transporters, tumors that exhibit graded metabolic heterogeneity contain cells expressing both MCT1 and MCT4 transports. In contrast, tumors expressing high levels of MCT4 do not favor metabolic symbiosis (10). However, the relation of the MCT1 and MCT4 expression on tumor cells and lactic acidosis in the microenvironment remains unclear.

Adenosine monophosphate-activated protein kinase (AMPK) regulates energy metabolism, but its participation in tumor metabolism remains uncertain. Yan et al. showed that loss of tumor suppressor folliculin (FLCN) induces the constitutive activation of AMPK, which results in peroxisome proliferator-activated receptor gamma coactivator 1-alpha (PGC-1α)-mediated mitochondrial biogenesis and increased ROS production. ROS induces hypoxia-inducible factor (HIF) transcriptional activity, driving the Warburg metabolic reprogramming. Thus, HIF can be indirectly activated by AMPK (13). Additionally, AMPK enhances glycolysis and glycolytic rate by directly phosphorylating 6-phosphofructo-2-kinase (PFK2) in H1299 tumor cells grown under glucose-limiting conditions (14). Conversely, inactivation of AMPK has been shown to increase HIF-1α expression, which in turn increases aerobic glycolysis and cellular biosynthesis in tumor cells. Thus, AMPK may be a negative regulator of the Warburg effect and suppresses tumor growth *in vivo* (15). Besides of hypoxia or AMPK inactivation, an acidic extracellular space also leads to the formation of a pseudo-hypoxic condition by increasing HIF function. Acidosis acts through HSP90, in a PHD/VHL-independent manner, to promote HIF function and maintenance of tumor stem cells in glioma (16, 17).

We hypothesized that if lung adenocarcinoma cells are in the presence of lactic acidosis with glucose availability, then tumor cells will perform the metabolic shift from aerobic glycolysis to OXPHOS, supported by AMPK activation.

## Materials and Methods

### Cell Lines

Three human tumor cell lines were used in this study. We included A-549 and A-427 cell lines, because they belong to the histological type of lung adenocarcinoma, which is the most prevalent subtype of lung carcinomas. MCF-7 cell line is a breast cancer cell line, it was included because it has been shown that can consume lactate in the absence of glucose (18). MRC-5 fibroblasts were included as control because they are proliferative non-transformed cells. All cell lines and fibroblast cells were obtained from the American Type Culture Collection (Manassas, VA, USA).

### Growth Curves

We used complete RPMI-1640 medium (Sigma-Aldrich, St. Louis, MO, USA) that contained 2 mM lactate and 10 mM glucose, it was supplemented with 10% heat-inactivated FCS (fetal calf serum, Hyclone, Logan, Utah, USA), 100 U/mL of penicillin and 100 μg/mL of streptomycin. Two 24-well plates were seeded equivalently. One plate was used for normoxic conditions, while the other was used for hypoxic conditions. A-427, A-549 and MCF-7 cells were seeded at a density of 1 × 10^5^ cells/mL, and 5 × 10^4^ cells/mL were seeded for MRC-5 cells.

Six wells of each plate were seeded with 1 mL of cellular suspension prepared in RPMI-1640 adjusted at pH 7.2. Other six wells of each plate were seeded with 1 mL of a cellular suspension prepared in RPMI-1640 adjusted at pH 6.2 using HCl (37% v/v). Normoxic cells were incubated in a humidified chamber at 37°C with filtered atmospheric air (21% oxygen) and 5% CO_2_. Hypoxic cells were incubated at 37°C, in a humidified Billups-Rothenberg chamber (Del Mar, CA, USA) with a gas atmosphere of 2% oxygen, 93% nitrogen, and 5% of CO_2_.

Every 8, 12, or 24 h, depending on the cell line and until completing 96 h, cell viability and cellular count were determined with trypan blue dye exclusion using a TC20 Automated Cell Counter (Bio-Rad Laboratories, Inc., USA). All cultures were repeated by triplicate. The specific growth rate (μ) was determined during exponential growth, as previously reported (19).

### Annexin V/7-AAD Assay

To determine viability, early apoptosis and necrosis of tumor cells and fibroblast cells, the method of apoptosis determination by Annexin V and 7-AAD was used. After 48 h of incubation, cells were harvested, washed with PBS and resuspended in 100 μL of Annexin V (0.5 μg/mL) (BioLegend, San Diego, CA, USA) in HEPES buffer. After incubation during 15 min, 7-aminoactinomycin D (7-AAD, BioLegend, 0.1 μg/mL) in HEPES buffer was added. Flow cytometry was performed using a Becton Dickinson FACSCanto II flow cytometer. The analysis was done using FlowJo V10 Software (Beckton Dickinson, Ashland, Or, USA).

### Cell Cycle Analysis

For cell cycle analysis, 1 × 10^5^ cells/mL of MRC-5, A-549, A-427, and MCF-7 cells were seeded per well in 24-well tissue culture plates. Cells were cultured during 48 h under the different conditions described above. Cells were harvested by trypsinization, washed with PBS, fixed using ethanol 70% v/v and permeabilized using Triton X-100 0.5 % v/v. Then, samples were stained with 7-AAD in the dark at room temperature for 20 min. A total of 25,000 events from 7-AAD-area versus 7-AAD-wide gate were acquired. Cultures and staining were independently performed at least two times.

### Determination of Glucose and Lactate

The supernatant from one well of the 24-well plates was removed every 8, 12, or 24 h, depending on the cell line and until completing 96 h. After measuring the volume, cell-free supernatants were stored at −20°C for subsequent analysis. A sample of the initial culture media was stored at −20°C and it was considered the time zero in the metabolites' analysis.

Using membranes that contain immobilized specific enzymes (d-glucose oxidase and l-lactate oxidase; YSI, Ohio, USA) and a YSI 2900 biochemistry analyzer (Yellow Springs Instruments, Ohio, USA), we determined the concentration of glucose and l-lactate in supernatants, as reported in (19). The evaporated volume was used to correct the quantity of each metabolite. Glucose consumption rate (q_SGlucose_) and lactate production rate (q_PLactate_) were calculated in the exponential phase of growth using the following formulas:

q_SGlucose_ = μ (h^−1^) ^*^ consumed glucose (μM) /cell number obtained during exponential phase ^*^ 1 × 10^6^

q_PLactate_ = μ (h^−1^) ^*^ produced lactate (μM) / cell number obtained during exponential phase ^*^ 1 × 10^6^

### Determination of GLUT1, GLUT4, MCT1 and MCT4 Expression on Cell Membrane

We used flow cytometry for determination of the expression of GLUT1, GLUT4, MCT1, and MCT4 on cancer and fibroblast cells. Cultures were initially seeded with 1 × 10^5^ cells/mL for A-549, A-427, and MCF-7 cell lines, 2 × 10^5^ cells/mL for A-427 and 1.5 × 10^5^ cells/mL for MRC-5 in 24-well plates. After 48 h of incubation, cells were harvested using EDTA-MOPS. Dead cells were excluded by using the Zombie NIR fixable viability kit (BioLegend, San Diego, CA, USA), as previously reported (19). After washing with PBS supplemented with albumin (1% w/v) and sodium azide (0.1% w/v), cells were immunostained with antibodies against GLUT1 (GLUT1-PE, clone 202915) from R&D Systems (Abingdon, UK), GLUT4 (GLUT4-Alexa 647) from Novus Biological (Abingdon, UK), MCT1 (MCT1-Alexa 647), and MCT4 (MCT4-Alexa 488) both from Bioss (Massachusetts, USA) incubating at room temperature for 30 min. Then cells were washed and fixed with paraformaldehyde (1% w/v) for further analysis using a FACS Canto II flow cytometer. At least 10,000 events were acquired from the region of viable cells. The results were analyzed with FlowJo V10 software. The median fluorescence intensity (MFI) values for GLUT1-PE, GLUT4-Alexa 647, MCT1-Alexa 647 and MCT4-Alexa 488 were determined. MFI values for the expression of GLUT1, GLUT4, MCT1 and MCT4 were normalized with respect to the condition of pH 7.2 and normoxia. The results are reported as relative MFI values (rMFI).

### Analysis of Mitochondrial Function

Mitochondrial function was evaluated measuring oxygen consumption using a Clark type electrode and the biological oxygen monitor system YSI 5300A (YSI incorporated, Yellow Springs, Ohio, USA). The reaction took place in a chamber with constant temperature of 37°C and constant stirring. The oxygen monitor system was calibrated according manufacturer's instructions.

After 48 h of incubation under the four tested conditions, lung adenocarcinoma cells (A-549 and A-427) were harvested by trypsinization. After a wash with PBS, cells were resuspended to obtain a cellular concentration of 1 × 10^6^ cells in 300 μL of respiration buffer. Respiration buffer contained KCl 120 mM, MgCl•6H2O 10 mM, EDTA 1 mM, KHPO_4_•7H_2_O 8.1 mM, K_2_HPO_4_ 1.46 mM, pH = 7.4 adjusted with KOH 10M. Cellular suspension (300 μL) was placed in the reaction chamber that already had 700 μL of oxygen-saturated respiration buffer at 37°C, in order to have 1 × 10^6^ cells/mL in the reaction chamber. The chamber was sealed and the oxygen concentration was registered each second using Hterm Software v. 0.8.1. In this way, basal respiration rate of tumor cells was obtained before digitonin addition (7.5 μg/mL) allowing cell permeabilization during 5 min. Then substrates and inhibitors of the respiratory chain where added to the reaction chamber in the following order and final concentrations: glutamate 10 mM, malate 5 mM, ADP/MgCl_2_ 1 mM, rotenone 10 μM, succinate 10 mM, antimycinA 10 μM as previously reported (20). The oxygen concentration in the reaction chamber was calibrated considering the oxygen-maximum saturation of the respiration buffer at atmospheric pressure of 585 mmHg at Mexico City at 37°C, which is 400 nM O_2_/mL. We obtained the oxygen consumption (nM O_2_/mL^*^1 × 10^6^ cells) versus time (min) plots. All the analyses of the mitochondrial function were made by tetraplicate.

### RNA Extraction

After 48 h of incubation under the conditions above mentioned, total RNA was isolated from tumor cells (A-549, A-427, MCF-7) and fibroblasts (MRC-5) using ZR-Duet DNA/RNA Miniprep according to manufacturer's instructions (Zymo Research, Irvine, CA, USA). Total RNA was treated with RNAse free DNAse I (Thermo Scientific, Waltham, MA, USA) according to the manufacturer's protocol. Quality and quantity of RNA were evaluated by A_260_ and A_280_ using NanoDrop 2000 (Thermo Scientific, Waltham, MA, USA). Total RNA was reverse-transcribed using the kit Maxima First Strand cDNA Synthesis Kit for RT-qPCR (Thermo Scientific, Waltham, MA, USA). The cDNA obtained was stored at−20°C for further analysis.

### Transcriptional Analysis of HIF-1α, AMPK and CS Using RT-qPCR

We used RT-qPCR to determine the transcript levels of HIF-1α, AMPK and CS. qPCR was performed in a semi-quantitative form using an ABI Prism 7500 Sequence Detector (Applied Biosystems, Foster City, CA). qPCR reaction contained SYBR Select Master Mix (Thermo Scientific, Waltham, MA, USA), cDNA as template and a pair of specific primers (HIF1α-F: 5′-CAG TCG ACA CAG CCT GGA T-3′ and HIF1α-R: 5′-TGG CAA GCA TCC TGT ACT GT-3′; AMPK-F: 5′-CAG GCC ATA CCC TTG ATG AAT-3′ and AMPK-R: 5′-TTC TTC CTT CGT ACA CGC AAA T-3′; CS-F: 5′- ACC TGT CAG CGA GAG TTT GC-3′ and CS-R: 5′- CCC AAA CAG GAC CGT GTA GT-3′. Validation curves were run using 18S rRNA, which was selected as endogenous control for all analyzed genes (18S-F: 5′-TAC CGC AGC TAG GAA TAA TGG-3′ and 18S-R: 5′- CGT CTT CGA ACC TCC GAC TT-3′).

PCR reactions were performed in 96-well reaction plates using the recommended parameters (10 min at 95°C, 40 cycles of 95°C for 15 s, and 60°C for 1 min.). Each PCR reaction was performed by triplicate and two non-template controls were included. Data were analyzed with Sequence Detection Software v 1.3.1 (Thermo Scientific, Waltham, MA, USA) to establish the PCR cycle at which the fluorescence exceeded a set of cycle threshold (Ct) for each sample. Comparative 2^−ΔΔCt^ method was used for target gene expression analysis (21). After normalization using the 18S rRNA housekeeping gene, normalized data of AMPK, HIF-1α, and CS expression from tumor and fibroblast cells were compared with the average of normalized data from same genes, expressed in tumor and fibroblast cells cultured in neutral pH media under normoxia. This condition was set as the value of 1 and was used as calibrator to compare data from the acidic conditions under normoxia or hypoxia.

### Analysis of HIF-1α, AMPK and pAMPK Protein Levels

HIF-1α, AMPK and pAMPK protein levels were determined in A-549, A-427, and MCF-7 tumor cell lines and MRC-5 fibroblast cells by flow cytometry. After 48 h of incubation under the tested conditions described above, HIF-1α and total AMPK were examined as previously reported (19). Briefly, cells were harvested by trypsinization, they were washed with PBS and stained with Zombie NIR. Then, cells were fixed and permeabilized with Transcription Factor Staining Buffer Set (Invitrogen) according to manufacturer's instructions. After permeabilization, cells were resuspended in 100 μL of PE mouse anti-HIF-1α monoclonal antibody (clone 546-16, BioLegend) or rabbit anti-AMPK alpha-1 polyclonal antibody (dilution 1:1000, cat. no. bs-10344R, Bioss Antibodies) for 45 min. In the case of AMPK staining, cells were further washed and incubated with Alexa 488 mouse anti-rabbit monoclonal antibody (Molecular Probes, Eugene Oregon) for 30 min. Finally, cells were washed and fixed using paraformaldehyde (1% w/v).

To evaluate the protein levels of phosphorylated form of AMPK, after incubation, cells were treated with Phosflow Fix Buffer I/Phosflow Perm Buffer III (BD Phosphoflow) according to manufacturer's protocol. Briefly, cells were detached and incubated with Phosflow Fix Buffer I at 37°C for 10 min. Then, cells were washed and incubated with Phosflow Perm Buffer III on ice for 30 min. After permeabilization, cells were washed with PBS/BSA and resuspended in 100 μL of rabbit anti-AMPK alpha-1/2 (Thr183/Thr172) polyclonal antibody (dilution 1:1000, cat. no. bs-4002R, Bioss Antibodies).

After 45 min of incubation with the primary antibody, cells were washed and incubated with Alexa 488 mouse anti-rabbit monoclonal antibody for 30 min. Cells were washed and resuspended in paraformaldehyde (1% w/v) to proceed with the flow cytometric analysis.

At least 10,000 events were acquired from the region of viable cells. The results were analyzed with FlowJo V10 software. The MFI values for HIF-1α, AMPK, and pAMPK were determined. Then the relative MFI (rMFI) was obtained using the formula rMFI= MFI treatment/MFI control. The control condition was pH7 and normoxia.

### Statistical Analysis

All values are expressed as the mean ± standard error. We used GraphPad Prism 7 software to test changes between groups. We usually established as the control group, the condition with the medium with lactate/glucose, pH7 and normoxia, and the differences between the control group and condition-tested groups were analyzed using unpaired Student's *T*-test. Significant differences between groups were considered at *p* < 0.05.

## Results

### Lactic Acidosis Differentially Affected Growth Rate of Tumor Cells

**Figure 1:**
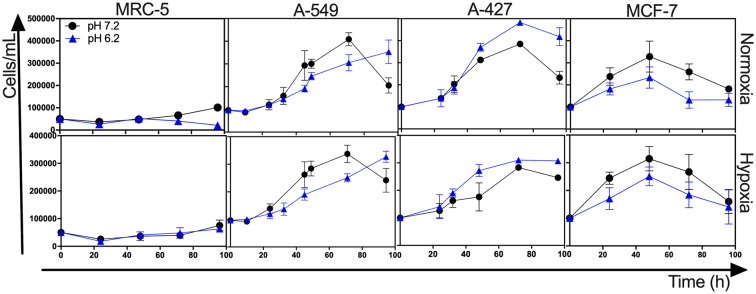
Growth curves of MRC-5 fibroblast cells and A-549, A-427, and MCF-7 tumor cells followed through 96 h, using RPMI-1640 glucose (10 mM) and lactate (2 mM) adjusted at pH 7.2 or pH 6.2 under normoxia (21% O_2_) or hypoxia (2% O_2_).

The initial concentrations of lactate and glucose in all cultures were 2 mM and 10 mM, respectively. All tumor cells consumed glucose and produced lactate during the exponential and stationary phases. As of 48 h of cell culture, lactate was accumulated in the extracellular medium reaching quantities up to 20 mM; thus, tumor and non-transformed cells created a lactic environment. Lactic acidosis (pH = 6.2) with the presence of glucose significantly increased the growth rate of A-427 tumor cells in comparison with neutral lactosis. Conversely, this same condition diminished the growth rate of A-549, MCF-7, and MRC-5 cells, significant differences were found under the normoxic condition (Figure 1, Table 1). Under hypoxia, only A-549 cells significantly diminished their growth rate when they were cultured under lactic acidosis in comparison with neutral lactosis (Table 1). As expected MRC-5 cells grew slower than tumor cells in all conditions tested in this study (Figure 1, Table 1). Remarkably, none of the tumor cell lines increased their growth rate under hypoxia in comparison with normoxia (Table 1).

**Table 1:** Specific growth rate of tumor cell lines and fibroblast cells.

	**Normoxia**		**Hypoxia**	
**Cell line**	**pH 7.2 (x10^**−2**^ h^**−1**^)**	**pH 6.2 (x10^**−2**^ h^**−1**^)**	**pH 7.2 (x10^**−2**^ h^**−1**^)**	**pH 6.2 (x10^**−2**^ h^**−1**^)**
MRC-5	1.1(0.3)	−0.4(0.3)^*^	0.8(0.4)	0.5(0.4)
A-549	2.3(0.2)	1.8(0.1)^*^	1.8(0.2)	1.5(0.1)^*^
A-427	1.9(0.2)	2.4(0.1)^*^	1.5(0.3)	1.4(0.2)
MCF-7	2.1(0.7)	1.6(0.6)	1.9(0.5)	1.8(0.5)

*Specific rate of growth was determined on exponential phase. All cultures were made by triplicate in tissue-culture plate using RPMI-1640 with glucose (10 mM) and lactate (2 mM) and pH 7.2 or pH 6.2 under normoxia or hypoxia. Values are expressed as mean (std error)*.

* *p < 0.05 difference with respect to normoxia pH 7.2 condition*.

**Figure 2:**
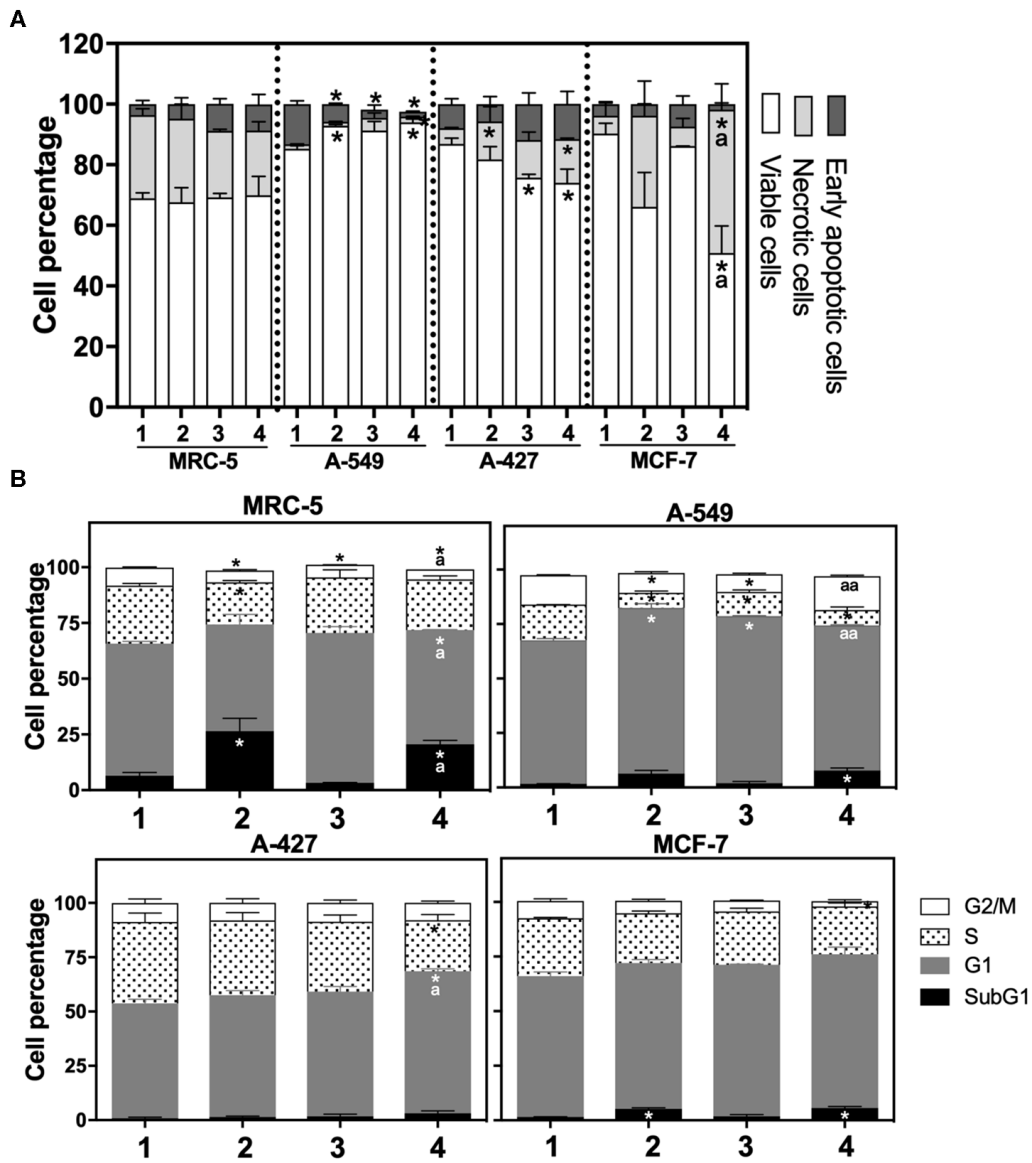
**(A)** Percentage of viability, necrosis and early apoptosis in lung cancer and fibroblast cells determined by AnnexinV/7-AAD assay. **(B)** Cell cycle profile of MRC-5 fibroblast cells, A-549, A-427, and MCF-7 tumor cells. (1) Normoxia, pH 7.2; (2) Normoxia, pH 6.2; (3) Hypoxia, pH 7.2 and (4) Hypoxia, pH 6.2. Bars represent mean+SEM. *p < 0.05 difference with respect to normoxia & pH7.2. ª*p* < 0.05, ªª*p* < 0.01 difference with respect to hypoxia and pH7.2.

After finding that lactic acidosis differentially affected tumor growth rate, we wanted to know how this condition affected cell viability and cell cycle progression in tumor and fibroblast cells. We found that, whereas the viability of MRC-5 and A-427 cells did not change, the viability of A-549 cells increased and the viability of MCF-7 cells diminished when these cells were cultured under lactic acidosis in comparison with neutral lactosis, either under normoxia or hypoxia (Figure 2A). Interestingly, the analysis of the cell cycle profile of MRC-5 cells showed that lactic acidosis significantly increased subG1 cell percentages and diminished G1 and S cell percentages in comparison with neutral lactosis (Figure 2B). In A-549 cell line, lactic acidosis significantly increased percentages of cells in G1-phase with a concomitant reduction in the percentages of cells in S-phase cell, in comparison with A-549 cells cultured under neutral lactosis (Figure 2B). A-549 and MCF-7 cells increased subG1 cell percentages under lactic acidosis irrespective of oxygen availability. Conversely, A-427 cells cultured under lactic acidosis did not modify the cell cycle profile in comparison with cells cultured under neutral lactosis (Figure 2B).

### Lactic Acidosis Diminished the Specific Rate of Lactate Production in Tumor Cells During the Exponential Growth Phase, but Only A-549 Cells Diminished the Specific Rate of Glucose Consumption

**Figure 3:**
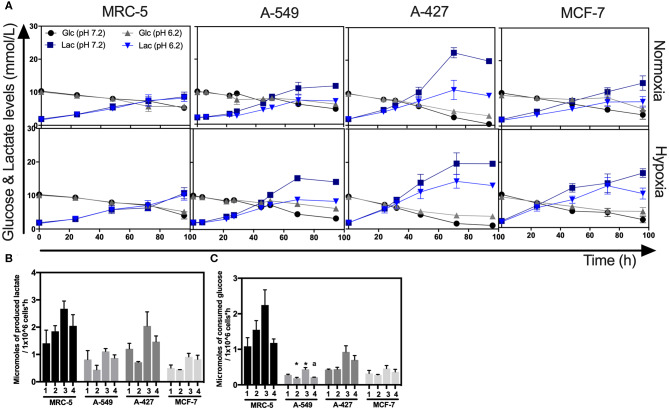
Glucose and lactate metabolism in tumor and fibroblast cells. **(A)** Kinetics of glucose and lactate levels from cultures of MRC-5, A-549, A-427, and MCF-7 followed through 96 h, using RPMI-1640 at pH 7.2 or pH 6.2 under normoxia (21% O_2_) or hypoxia (2% O_2_). **(B)** Specific rate of lactate production obtained during exponential phase of growth of tumor and fibroblast cells. **(C)** Specific rate of glucose consumption obtained during exponential phase of growth of tumor and fibroblast cells. Bars represent mean + SEM. *p < 0.05 difference with respect to normoxia and pH7.2. ª*p* < 0.05 difference with respect to hypoxia and pH7.2.

As above indicated, all cell lines consumed glucose and produced high amounts of lactate. At the end of the culture, we found that A-427 cell line consumed more glucose and produced more lactate than A-549, MCF-7 and MRC-5 cells under the four tested conditions (Figure 3A). None of the cell lines (tumor cells and non-transformed cells) consumed lactate under lactic acidosis independently of the oxygen concentration; instead, all cell lines consumed glucose (Figure 3A). But at the end of 96 h of cell culture, when glucose levels were very low, A-549, A-427, and MCF-7 cells consumed lactate; as opposed to MCR-5 fibroblast cells, which did not consume lactate throughout the culture (Figure 3A).

During exponential phase, the specific rate of lactate production (q_PLactate_) of tumor and fibroblast cells tended to diminish when cells were cultured under lactic acidosis in comparison with neutral conditions under normoxia or hypoxia (Figure 3B). As it was expected, under hypoxia all tumor and fibroblast cells tended to increase q_PLactate_ in comparison with normoxia regardless of the pH (Figure 3B).

When tumor cells were cultured under acidosis compared with neutral conditions, the specific rate of glucose consumption (q_SGlucose_) of A-427 and MCF-7 tumor cells and MCR-5 cells did not change during exponential phase (Figure 3C); in contrast, the q_SGlucose_ of A-549 cells significantly diminished (Figure 3C). As expected, under hypoxia all tumor and fibroblast cells increased their specific rate of glucose consumption (q_PGlucose_) in comparison with normoxia (Figure 3C).

### Lactic Acidosis Diminished GLUT1 and GLUT4 Expression in A-549 Cells, but Not in A-427 Cells

**Figure 4:**
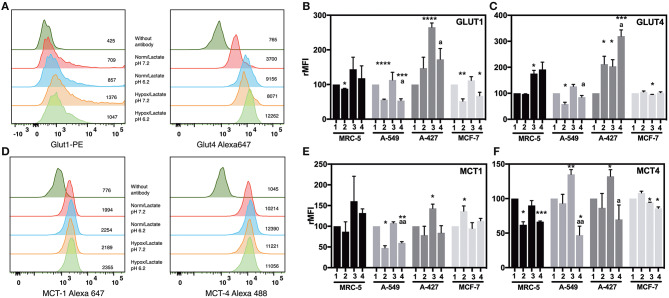
Analysis of the expression of glucose and monocarboxylate transporters on tumor and fibroblast cells. **(A)** Histograms of a representative experiment of GLUT1 and 4 and **(D)** MCT1 and 4 expression analysis on A-427 tumor cell line. Analysis was performed on Zombie-NIR low (viable) cells and MFI values for each treatment are shown. Relative media fluorescence intensity (rMFI) of **(B)** GLUT1 and **(C)** GLUT4 on tumor and fibroblast cells. Relative media fluorescence intensity (rMFI) of **(E)** MCT1 and **(F)** MCT4 on tumor and fibroblast cells. Bars represent data of at least two independent culture experiments expressed in mean + SEM. *p < 0.05, **p < 0.01, ***p < 0.001, ****p < 0.0001 difference with respect to normoxia and pH7.2. ª*p* < 0.05, ªª*p* < 0.01 difference with respect to hypoxia and pH7.2.

To know the effect of acidosis on glucose transporters expression, we evaluated GLUT1 and GLUT4 expression on tumor cells after 48 h of incubation. Representative flow cytometric analyses for GLUT1 and GLUT4 of A-427, MRC-5, A-549, and MCF-7 cells are shown in (Figure 4A and Supplementary Figure 1).

Lactic acidosis increased GLUT1 levels in A-427 cells in comparison with neutral lactosis under normoxia. In contrast, lactic acidosis significantly diminished GLUT1 expression levels in A-549, MCF-7, and MRC-5 cells in comparison with neutral conditions under both normoxia and hypoxia (Figure 4B). Contrary to A-549 cells, lactic acidosis increased GLUT4 expression levels in A-427 cells, compared with neutral conditions under normoxia or hypoxia. Interestingly, hypoxia increased GLUT4 expression levels in A-427 and MCR-5 cells in comparison with normoxia (Figure 4C).

Additionally, it has been reported that hypoxia can increase glucose consumption (11, 12). Accordingly, we found that hypoxia tended to increase GLUT1 and GLUT4 expressions in A-549, A-427 and MCF-7 tumor cells and MCR-5 fibroblast cells; but only A-427 cells presented significant differences, and MCF-7 did not increase GLUT4 under hypoxia (Figures 4B,C).

### Lactic Acidosis Diminished the Expression of Both Monocarboxylate Transporters MCT1 and MCT4 in Lung Adenocarcinoma Cells

Regarding lactate metabolism, none of cell lines consumed lactate during the exponential phase of the growth curve, lactate concentrations were always increasing in the media up to 72 h of culture (Figure 3A). To corroborate this finding, we evaluated MCT1 and MCT4 expression on tumor cells after 48 h of incubation. Representative flow cytometric analyses for MCT1 and MCT4 of A-427 MRC-5, A-549 and MCF-7 cells are shown in (Figure 4D and Supplementary Figure 1). We found that under lactic acidosis, A-427 and A-549 tumor cells and MRC-5 cells diminished MCT1 levels with respect to neutral conditions independently of the oxygen tension, although only A-549 cells presented significant differences. In contrast, MCF-7 cells significantly increased MCT1 expression only when these cells were cultured under normoxia (Figure 4E).

When we analyzed MCT4 expression, we found that lactic acidosis also diminished MCT4 expression in A-427 and A-549 tumors cells and MRC-5 fibroblast cells (Figure 4F); these data corroborate our previous result where lactic acidosis tended to diminish q_PLactate_ of tumor cell lines in comparison with neutral conditions (Figure 3B). In contrast, MCF-7 cells tended to increase MCT4 expression under lactic acidosis and normoxia (Figure 4F). Interestingly, under neutral lactosis and hypoxia, A-549 and A-427 lung cancer cells increased MCT4 expression in comparison with normoxia (Figure 4F), which correlates with an increased q_PLactate_ in hypoxia with respect to normoxia (Figure 3B).

### Under Lactic Acidosis, Mitochondrial Respiration of A-427 Cells Was Increased but Mitochondrial Respiration of A-549 Cells Was Diminished

After finding that lactic acidosis diminished lactate production by tumor cells, we evaluated the effect of acidosis on mitochondrial functionality in lung tumor cells; thus, we determined the oxygen consumption in A-549 and A-427 cells using a Clark-type electrode.

We found that A-549 cells consumed more oxygen per minute than A-427 cells under both conditions: lactic acidosis and neutral lactosis (see Basal respiration in Table 2). However, in A-549 cells cultured under lactic acidosis, the oxygen consumption per minute significantly diminished in comparison with A-549 cells cultured under neutral conditions. In contrast, the basal respiration rate of A-427 cells cultured under lactic acidosis increased in comparison with A-427 cells that were cultured under neutral lactosis (Table 2). Interestingly, the respiratory state III or maximal respiration state, obtained after adding glutamate, malate and ADP, was higher in A-427 cells cultured under lactic acidosis than cells cultured under neutral lactosis (Table 2).

**Table 2:** Evaluation of mitochondrial respiration of A-549 and A-427 tumor cells using different substrates and inhibitors of the respiratory chain.

**Cell line**	**A-549**		**A-427**	
**Media**	**pH 7.2**	**pH 6.2**	**pH 7.2**	**pH 6.2**
Basal respiration	1.8 (0.8)	0.72 (0.13)^*^	0.3(0.15)	0.5(0.005)^*^
Glutamate/Malate/ADP	0.6 (0.3)	0.6(0.3)	0.13(0.002)	0.5(0.12)^*^
Rotenone	−0.13(0.01)	−1.5(0.3)	−0.3(0.01)	−0.3(0.01)
Succinate	0.38(0.15)	0.7(0.1)^*^	0.17(0.05)	0.5(0.3)^*^
AntimycinA	−0.24(0.01)	−0.4(0.05)^*^	−0.06(0.01)	−0.21(0.1)^*^

*Oxygen consumption rate (nM consumed oxygen/min × 10^6^ cells) was determined on tumor cells after 48 h of incubation under normoxia. Experiments were made by tetraplicate. Values are expressed as mean (std dev)*.

* *p < 0.05 difference with respect to normoxia pH 7.2 condition*.

When we added succinate, the substrate for the complex III of OXPHOS, the oxygen consumption rate was significantly higher in tumor cells cultured under lactic acidosis (Table 2). Thus, the complex III of both tumor cell lines was more active when these cell lines were cultured under lactic acidosis.

### The Differential Expression of HIF-1α, AMPK and CS Transcript Levels Correlated With the Energetic Metabolism of Tumor Cells Under Lactic Acidosis

To figure out how the metabolism of tumor and fibroblast cells was regulated under lactic acidosis, we evaluated AMPK, HIF-1α, and CS transcript levels on these cells after 48 h of incubation under the four culture conditions.

**Figure 5:**
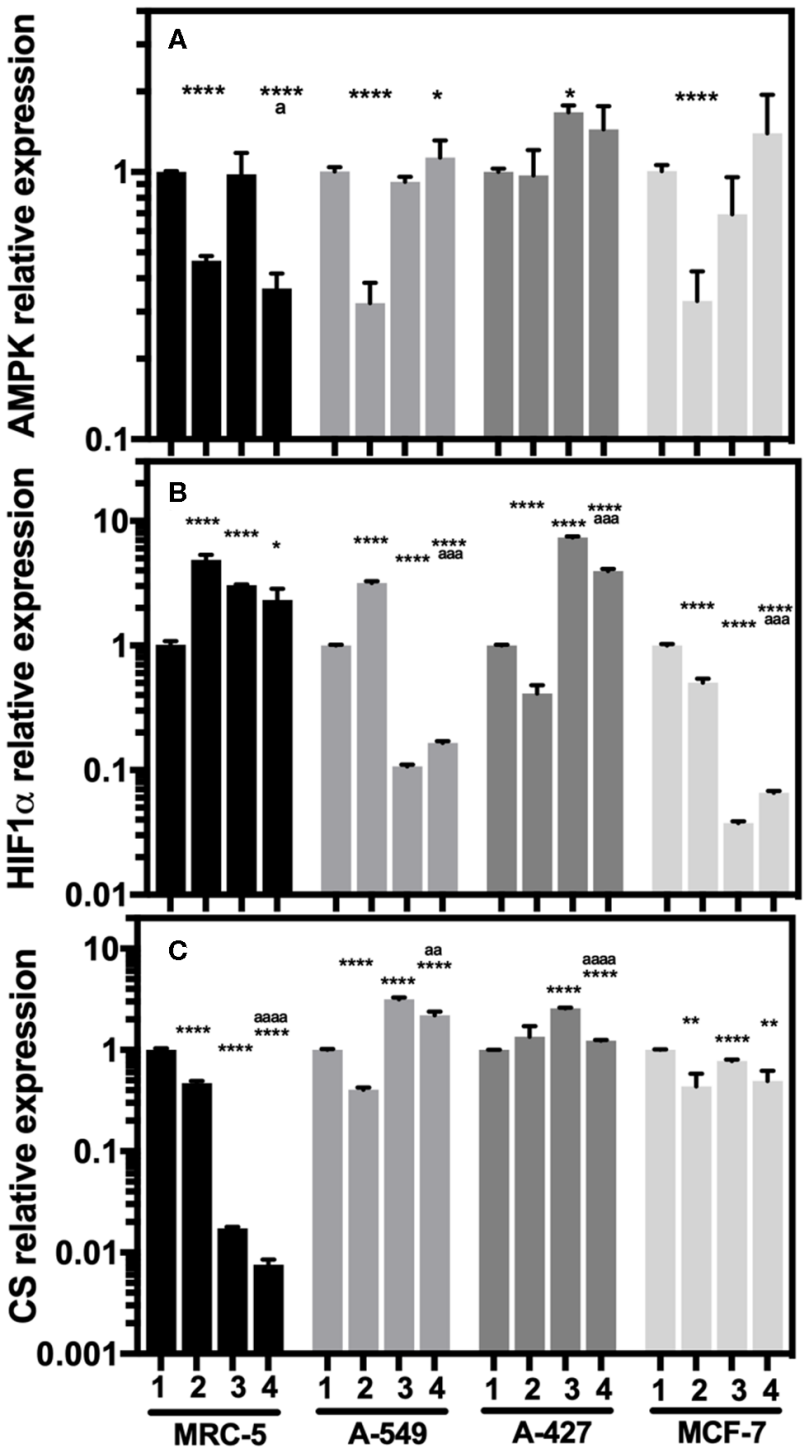
Relative quantification of AMPK, HIF-1α, and CS transcript levels in cancer cell lines and fibroblasts cultured for 48 h. **(A)** AMPK, **(B)** HIF-1α, and **(C)** CS relative expression in the tumor cells and fibroblast cultured under lactic acidosis with respect to neutral lactosis under normoxia, this condition was set as the value of 1. (1) Normoxia, pH 7.2; (2) Normoxia, pH 6.2; (3) Hypoxia, pH 7.2 and (4) Hypoxia, pH 6.2. Bars represent transcriptional data of two independent culture experiments expressed in mean+SEM. *p < 0.05, **p < 0.01, ****p < 0.0001 difference with respect to normoxia and pH7.2. ª*p* < 0.05, ªªª*p* < 0.001, ªªªª*p* < 0.0001 difference with respect to hypoxia and pH 7.2.

When we compared the transcript levels of tumor cells grown under lactic acidosis in comparison with neutral lactosis, we found that A-549 and MCF-7 tumor cells and MRC-5 fibroblast cells significantly diminished AMPK transcript levels under normoxia (Figure 5A). These cells also presented a significant increase of HIF-1α transcript levels and a significant decrease of CS expression under normoxia (Figures 5B,C). These data suggest that mitochondrial activity is diminished in A-549 and MCF-7 tumor cells and MRC-5 fibroblast cells cultured under lactic acidosis and normoxia. Interestingly, we found a strong inhibition of CS expression in MRC-5, but not in tumor cells, cultured under hypoxia independent of pH (Figure 5C). In contrast, when we compared the transcript levels of A-427 tumor cells grown under lactic acidosis in comparison with neutral lactosis, we found that A-427 cells did not change the AMPK transcript levels under normoxia (Figure 5A). This phenomenon was accompanied by a significant reduction of HIF-1α transcript levels and an increase of CS expression (Figures 5B,C). Taken together our data indicate that mitochondrial activity is increased in A-427 cells cultured under lactic acidosis and normoxia.

Additionally, hypoxia significantly increased HIF-1α transcript levels in MRC-5 fibroblast cells and A-427 tumor cells (Figure 5B). This result correlated with the increase of GLUT1 expression in MRC-5 and A-427 cells under hypoxia compared with normoxia (Figure 4B).

### Protein Analysis of HIF-1α, AMPK and pAMPK Correlated With the Energetic Metabolism of Adenocarcinoma Cells Cultured Under Lactic Acidosis

**Figure 6:**
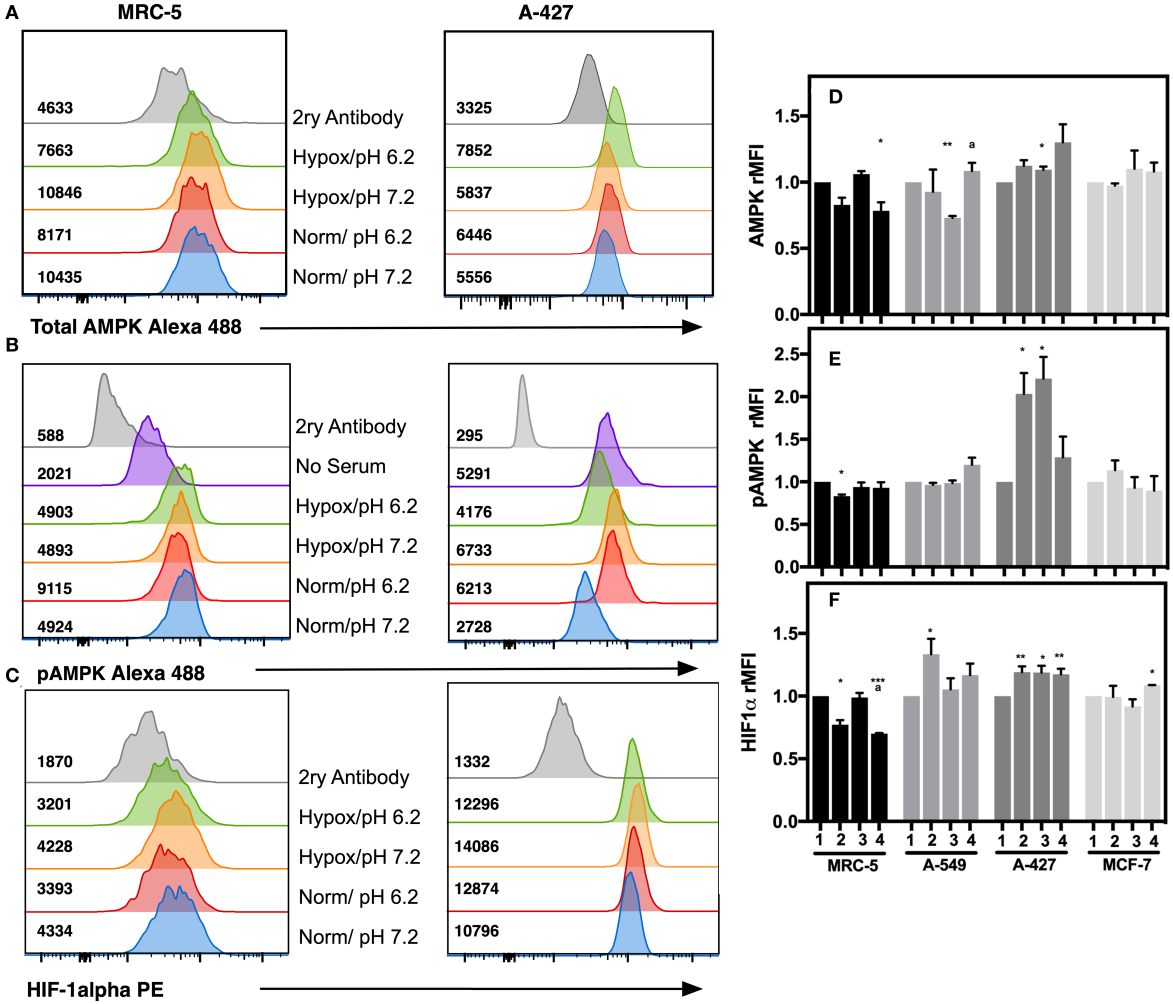
Determination of intracellular protein levels of total and phosphorylated AMPK, and HIF-1α in cancer cell lines and fibroblasts cultured for 48h. Histograms of a representative experiment for the determination of **(A)** AMPK, **(B)** pAMPK, and **(C)** HIF-1α proteins on MCR-5 fibroblast cells and A-427 adenocarcinoma cells, MFI values for each treatment are shown. rMFI of **(D)** AMPK, **(E)** pAMPK, and **(F)** HIF-1α intracellular protein levels in fibroblast and tumor cells cultured during 48 h under (1) Normoxia, pH 7.2; (2) Normoxia, pH 6.2; (3) Hypoxia, pH 7.2 and (4) Hypoxia, pH 6.2. Bars represent data of two independent experiments expressed in mean + SEM. *p < 0.05, **p < 0.01, ***p < 0.001 difference with respect to normoxia and pH7.2. ª*p* < 0.05, difference with respect to hypoxia and pH 7.2.

To corroborate the transcriptional analysis, we evaluated the protein levels of total and phosphorylated AMPK (pAMPK), as well as HIF-1α in tumor and fibroblast cells cultured under the analyzed conditions after 48 h incubation. Representative flow cytometric analyses for AMPK, pAMPK, and HIF-1alpha are shown in (Figures 6A–C). For the determination of the phosphorylated form of AMPK, we included as control serum-starved cells (FCS-free RPMI-1640 medium), because this condition lowers basal phosphorylation levels. We found that in MCR-5 cells, MFI values for pAMPK were the lowest and consequently presented the less phosphorylated state, followed by MCF-7 cells and A-549 cells, being rMFI values 0.39, 0.87, and 0.90, respectively. Interestingly, A-427 cells presented the highest levels of pAMPK (rMFI = 1.77). A similar phenomenon has been reported in L6 myotubes, which respond to insulin and, under serum starvation, increase the levels of pAMPK and GLUT4, leading to increase of glucose uptake (22). Interestingly A-427 was the only cell line where GLUT4 expression levels responded to changes in the microenvironment (see Figure 4C).

We also found that under lactic acidosis, MRC-5 cells diminished total AMPK and HIF-1α levels independent of oxygen tension (Figures 6D,F). Lactic acidosis diminished pAMPK levels only when cells were cultured under normoxia (Figure 6E). These results correlated with the transcript levels in MRC-5 cells for AMPK but not with HIF-1α (Figures 5A,B).

A-549 cells cultured under lactic acidosis or hypoxia increased HIF-1α protein levels, but pAMPK levels did not change in comparison with neutral lactosis and normoxia (Figures 6E,F).

A-427 cells cultured under lactic acidosis or hypoxia increased total and pAMPK levels, as well as HIF-1α protein levels in comparison with neutral lactosis and normoxia (Figure 6). These results correlated with the transcript analysis of A-427 cells cultured under hypoxia, where AMPK and HIF-1α mRNA levels increased (Figures 5A,B).

MCF-7 cells cultured under lactic acidosis or hypoxia did not change total and phosphorylated AMPK, nor HIF-1α protein levels in comparison with neutral lactosis and normoxia (Figure 6). These results correlated with the transcript analysis of MCF-7 cells cultured under lactic acidosis and hypoxia, where the AMPK and HIF-1α mRNA levels did not change (Figures 5A,B).

## Discussion

Because lactic acidosis with glucose availability is a frequent condition found in solid tumors, we wanted to investigate if tumor cells fulfilled the metabolic shift under lactic acidosis and normoxic or hypoxic conditions. Lactic acidosis under normoxia or hypoxia affected differentially the growth, glucose and lactate transporters expression, glucose consumption and lactate production, as well as mitochondrial functionality of both lung tumor cell lines (A-549 and A-427), in spite of both being adenocarcinomas.

Our data support that there is a tumor-metabolic heterogeneity, because cell lines like A-549 and MCF-7 cultured under control conditions exhibited a more oxidative metabolism, evidenced by a higher O_2_ consumption, while A-427 cells presented a more glycolytic metabolism because they consumed less oxygen per minute. Nonetheless, A-427 tumor cells were more adaptable to an acidic microenvironment, because they could shift to a more oxidative metabolism.

These results correlated with the heterogeneous metabolic phenotypes found in solid lung tumors *in vivo* (23). A-427 cells cultured under lactic acidosis and normoxia increased their growth rate, possibly supported by the increased expression of both the ubiquitously and the insulin-responsive glucose transporters (GLUT1 and GLUT4).

To our knowledge this is the first report that shows that lactic acidosis can induce GLUT4 expression in some tumor cells under normoxia or hypoxia.

AMPK activation may indirectly increase the expression of the glucose transporters. Although A-427 cultured under lactic acidosis and normoxia maintained the same AMPK transcript levels, total AMPK protein levels were slightly augmented, while AMPK activation was increased, as evidenced by higher levels of phosphorylation. In this regard, it has been reported that AMPK activation upregulates energy-producing catabolic processes, including glycolysis through GLUT1, GLUT4, HK, and PFK2 upregulation, as well as fatty acid oxidation induced by downregulating acetyl-coA carboxylase 2 (24). Also, Ching et al. previously showed that activation of AMPK under serum starvation conditions is required for glucose transport mediated by GLUT4 (22). Thus our data suggest that some lung tumor cells might exhibit a response to lactic acidosis similar to that presented by myotubes under serum starvation. The diminished mRNA levels of HIF-1α correlated with an increased expression of citrate synthase, alongside with the increase of mitochondrial respiration rate in A-427 cells cultured under lactic acidosis and normoxia; nonetheless, HIF-1α protein levels presented a slight increase. This apparent inconsistency could be caused by the stress-induced chaperone protein HSP390, which has been reported to interact with HIF-1α, protecting it from proteasomal degradation (16).

Tumor cells cultured under lactic acidosis exhibit an energetic metabolic shift for obtaining energy, first by aerobic glycolysis and then by OXPHOS (7). Remarkably, we now report that this phenomenon is mediated by an increase in the activity of the OXPHOS complexes I and III in A-427 cells. Taken together, these results indicate that lactic acidosis promoted the metabolic shift from aerobic glycolysis to oxidative metabolism in A-427 lung adenocarcinoma cells. This change might be advantageous because these tumor cells increased the growth rate under lactic acidosis, whereas MRC-5 non-transformed cells increased the percentage of dead cells under this same condition.

On the other hand, the growth rate of A-549 cells diminished when these cells were cultured under lactic acidosis regardless of oxygen tension. This result correlated with the decreased expression of both glucose transporters. Although under lactic acidosis and normoxia AMPK transcript levels were reduced, neither the protein nor activated AMPK levels changed. This possibly allowed the increase of HIF-1α at transcript and protein levels, as it has been observed that AMPKα deletion promotes elevated HIF-1α protein levels in two different tumor cell lines under normoxia (15). Our results suggest that the increased expression of HIF-1α promoted the decrease in citrate synthase expression, which correlated with the reduced respiration rate.

It has been reported that hypoxia-mediated HIF-1α upregulation increases the levels of GLUT1 (25); however, A-549 cells cultured under lactic acidosis downregulated the levels of both glucose transporters. These results agree with those previously reported by Giatromanolaki et al. who indicated that acidosis can eliminate the hypoxia-induced expression of glucose transporters (11); moreover, it has been reported that inhibition of AMPK and CaMKK (calcium/calmodulin-dependent protein kinase) inhibits GLUT4 translocation (26).

Taken together, our results indicate that in A-549 cells, lactic acidosis diminished Warburg effect, because lactate levels and lactate production rate were lower than in cells cultured under neutral lactosis. Importantly, although A-549 cells diminished Warburg effect, they did not shifted their metabolism, because respiration rate did not increase. Interestingly, even when A-549 cells were cultured under lactic acidosis and with glucose availability, these cells diminished their growth, but they did not die. Instead, A-549 cells maintained viability by promoting an arrest in G1-phase. A similar phenomenon has been observed in breast tumor cells (4T1), which induce G1 arrest, autophagy and inhibit apoptosis as survival mechanisms to protect cells from glucose deprivation-induced death when they are cultured under lactic acidosis (27).

We found that the non-transformed MRC-5 cells cultured under neutral lactosis and normoxia produced up to 10 mM of lactate, they also presented higher CS transcript levels under normoxia in comparison with hypoxia. Thus, MRC-5 fibroblast cells cultured under neutral lactosis and normoxia used both OXPHOS and aerobic glycolysis for obtaining energy. MRC-5 cells cultured under lactic acidosis and normoxia or hypoxia diminished their growth rate, the expression of GLUT1, MCT1 and MCT4, the transcript levels of AMPK and CS, as well as the protein levels of AMPK and HIF-1α in comparison with neutral lactosis, though only under normoxia the differences were significant. Under lactic acidosis and normoxic conditions the reduced expression of GLUT1 and monocarboxylate transporters was possibly promoted by the reduction of both HIF-1α and AMPK protein levels. Additionally, lactic acidosis inhibited cell cycle progression in MRC-5 fibroblast by reducing the transition of cells from G1-phase to S-phase, which correlated with the reduction of cell growth rate, and the increase in cell death. Thus, lactic acidosis might be a deleterious condition for proliferative non-transformed cells.

Wu et al. reported that lactic acidosis promoted a metabolic shift from aerobic glycolysis to OXPHOS, because the protons of intracellular acidosis inhibit the activity of some glycolytic enzymes (7). Our results partially support the findings of Wu et al., as we found that lactic acidosis diminished Warburg effect in lung adenocarcinoma cells, because they diminished lactate production rate, lactate levels and MCT1 and MCT4 expression under this condition. However, we did not find that reduction of aerobic glycolysis necessarily shifts the metabolism into OXPHOS; in view that although A-549 cells diminished aerobic glycolysis, they did not increase OXPHOS. In fact, A-549 cells cultured under lactic acidosis diminished their basal respiration rate.

Tumor cells that in the presence of glucose and normoxia opt to consume lactate rather than glucose might support tumor development by two mechanisms: tumor metabolic symbiosis (10, 28) and reverse Warburg effect (29).

A recent report suggests that non-glucose nutrients have potential contributions in well-perfused tumor areas *in vivo*, but some human lung tumor may use lactate as a carbon source (30). We found that none of the tumor cells consumed lactate during the exponential phase under the tested conditions. On the contrary, under lactic acidosis and regardless of oxygen tension, adenocarcinoma cells and fibroblast cells diminished MCT1 expression after 48 h of culture. However, when the three tumor cells reached the end of the culture (96 h) and levels of glucose were about 4 mM, they consumed small quantities of lactate, a phenomenon that did not occur in non-transformed cells.

Thus, our results suggest that tumor cells might participate in the metabolic symbiosis phenomenon when they are not in the exponential phase of cell growth. Although lactic acidosis may diminish glucose transporter expression in some adenocarcinoma cells, if they have glucose availability, they will continue consuming glucose during the exponential phase. Additionally, some adenocarcinoma cells cultured under lactic acidosis shifted their metabolism from Warburg effect to OXPHOS, because they increased their basal respiration rate which may be supported by the strong activation of AMPK.

## Data Availability Statement

The datasets generated for this study are available upon reasonable request from the corresponding author.

## Author Contributions

SR-G designed the study. SR-G, AC-H, and HP-G performed the experiments. HP-G and SR-G wrote and critically reviewed the manuscript. All authors contributed to manuscript revision, read and approved the submitted version.

## Conflict of Interest

The authors declare that the research was conducted in the absence of any commercial or financial relationships that could be construed as a potential conflict of interest.
