# Supplementary figures and images for: Lactic Acidosis in the Presence of Glucose Diminishes Warburg Effect in Lung Adenocarcinoma Cells
Source: Front Oncol. 2020 Jun 12;10:807. doi: 10.3389/fonc.2020.00807 (PMC7303336; doi:10.3389/fonc.2020.00807)

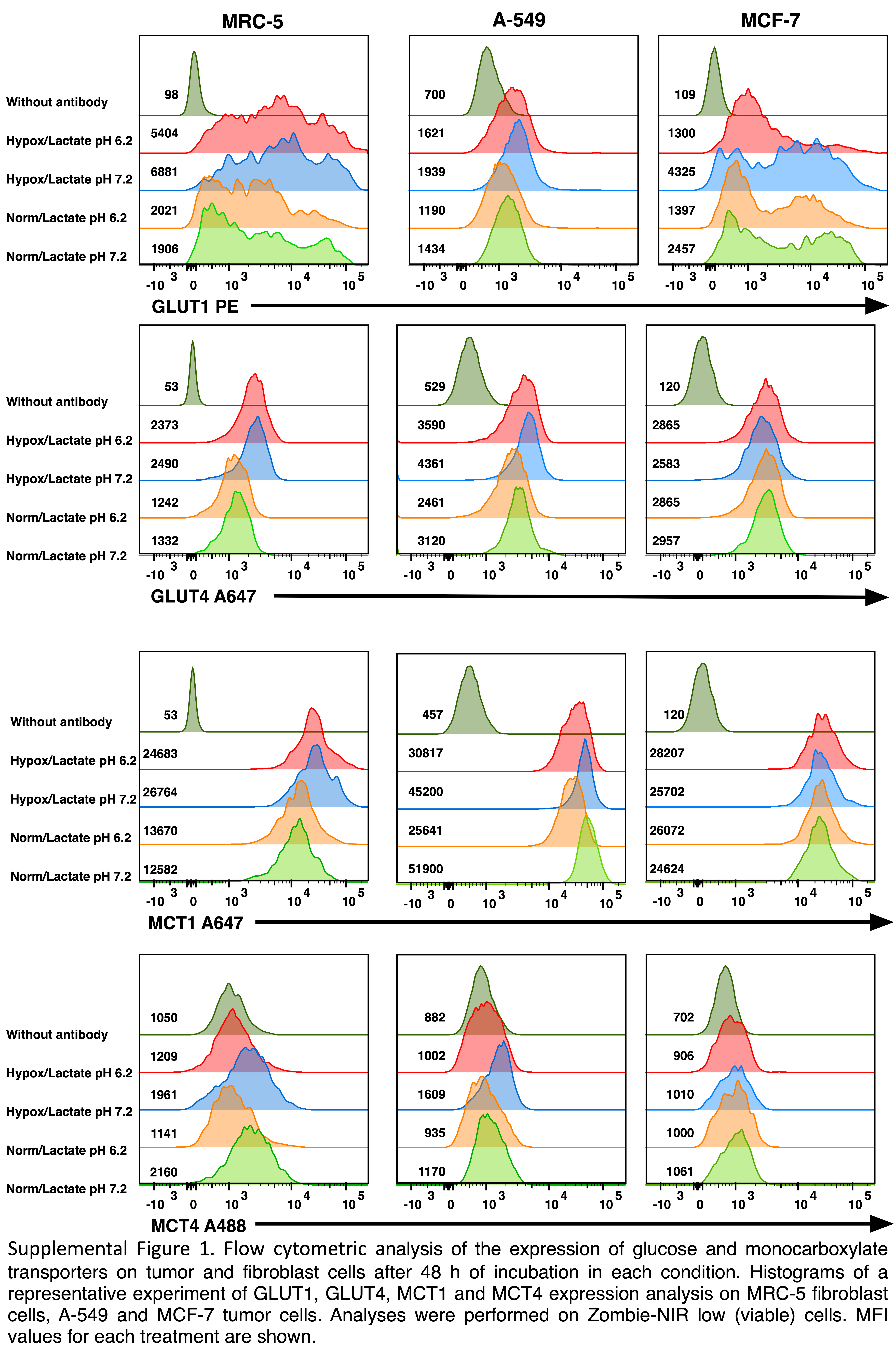

Supplement: Supplementary file 1 [file Image_1.TIFF]
